# Dendritic cell immunoreceptor (DCIR) is associated with anti-cyclic citrullinated peptides (anti-CCP) antibody - negative rheumatoid arthritis in Chinese Han population

**DOI:** 10.1186/ar3623

**Published:** 2012-02-09

**Authors:** Jianping Guo, Xinyu Wu, Xiaolan Lu, Yin Su, Ru Li, Jing He, Xu Liu, Zhanguo Li

**Affiliations:** 1 Department of Rheumatology and Immunology, Peking University People's Hospital, Beijing, 100044, China

## Background

The dendritic cell immunoreceptor (DCIR) is an important member of C-type lectin superfamily, which has been shown evidence for susceptibility to arthritis in multiple animal models. The human DCIR polymorphisms have been shown a nominal association with rheumatoid arthritis (RA) susceptibility, mainly with anti-cyclic citrullinated peptides (anti-CCP) antibody -negative RA in Swedish population. We aimed to investigate the possible association of *DCIR *with RA susceptibility in Chinese Han population.

## Methods

A total of 1193 patients with RA and 1278 healthy controls were genotyped for single-nucleotide polymorphism (SNP) rs2377422 and rs10840759. Association analyses were performed on the whole data set and on RA subsets based on the status of anti-CCP antibody in RA patients. The interaction between rs2377422 and HLA-DRB1 shared epitope (SE) was also analyzed for RA susceptibility. Finally, we carried out association analysis of rs2377422 with DCIR mRNA expression in RA patients.

## Results

The DCIR rs2377422 was found significantly associated with RA (allele analysis: OR 1.17, 95%CI 1.04-1.31, *p=*3.67 × 10^-3^; genotype analysis (recessive model C/C vs. T/T + T/C): OR 1.37, 95%CI 1.08-1.73, *p=*9.04 × 10^-3^). Following stratification for anti-CCP status, a suggestive association of rs2377422 with anti-CCP-positive RA was observed (*p = *0.058, OR 1.34, 95%CI 0.99-1.82). In contrast, the CC genotype of rs2377422 was found specifically to confer susceptible risk for anti-CCP-negative RA (OR 1.92, 95%CI 1.27-2.90, *p=*1.99 × 10^-3^), despite loss of power in the analysis. The relative risk of RA was 3.0 (95%CI 1.33-6.91, *p=*6.48 × 10^-3^) in individuals carrying rs2377422 TT genotype with SE alleles, and 9.06 (95%CI 3.33-25.61, *p=*2.08 × 10^-6^) in individuals carrying rs2377422 CC genotype with SE genes. The interaction between rs2377422 and SE alleles was significant, as measured by the attributable proportion (AP) due to interaction (0.60). DCIR gene transcription quantification analysis further proved the dominant effect of rs2480256 CC genotype on DCIR expression levels in RA patients (C/C vs. T/T + T/C: 0.55 ± 0.09 vs. 0.24 ± 0.02, *p=*1.67 × 10^-3^).

## Conclusions

Our study provides evidence for association between DCIR rs2377422 and RA, particularly with anti-CCP-negative RA in non-Caucasian populations.

